# Among Missouri Valley Veterinarians

**Published:** 1898-08

**Authors:** 


### AMONG MISSOURI VALLEY VETERINARIANS.

Dr. W. B. Niles, of Ames, Iowa, will take charge of the serum-treatment experimental work relative to hog-cholera in Iowa.

Dr. Oscar Verschelden, of St. Mary's, Kan., is sojourning in Europe for a period.

The inspection service at South St. Joseph, Mo., originally was composed of Drs. John M. Forbes, James Wilson, and James S. Kelly. Recently there have been added to the force at this point Drs. Howard M. Burgess, Thomas H. Ripley, H. J. Washburn, John B. Wright, and W. W. Johnston.

Dr. Harry V. Good, of St. Joseph, has sold his practice to Dr. J. Netherton, of Gallatin, Mo.

Drs. T. J. Turner, of Indianapolis, Ind.; I. K. Atherton, of Marshalltown, Ia.; E. P. Schaffter, of Cleveland, O.; N. R. Thompson, of Pueblo, Col.; F. W. Hopkins, of E. St. Louis, Ill.; and T. A. Bray, of El Paso, Tex., have received honorary membership in the Missouri Valley Veterinary Medical Association.

Drs. S. Stewart, W. A. Heck, and John Forbes are moving spirits in the Missouri Valley Veterinary Medical Association.

Dr. J. E. Blackwell, of South Omaha, Neb., has been transferred to the inspection service at South St. Joseph, and Dr. W. A. Heck, of Kansas City, has been transferred to the same point and placed in charge of the microscopic inspection.

Dr. W. N. D. Bird, of the class of 1898, Kansas City Veterinary College, has charge of the quarantine service at Arkansas City, Kan. He recently made a tour of the Wichita, Kiowa and Comanche reservations looking up the existence and extent of Texas fever, which has been somewhat prevalent in these districts.

Dr. C. H. Davies, M.D., D.V.S., another of the class of 1898 of the Kansas City Veterinary College, is stationed at Swift's establishment, Kansas City, in the meat-inspection service; Dr. John D. Cooper at Kansas City and W. W. Johnson, M.D., V.S., at St. Joseph, Mo.

James Otterman, M.D., D.V.S., and Dr. W. N. D. Bird will be transferred to Louisville, Ky., on September 1st.

Dr. Charles J. Sihler, of Kansas City, Mo., has been reappointed assistant inspector and assigned to duty at Monett, Mo., in charge of cattle-transportation on the St. Louis and San Francisco Railway.

Dr. Sylvester J. Murray, of Medo, Minn., graduate of Toronto, class of 1894, has been appointed an inspector in the Bureau of Animal Industry, and is stationed at Parsons, Kan., on the Missouri, Kansas, and Texas Railway in quarantine-line service duty.

Colonel Albert Dean, livestock agent at Kansas City, has been transferred to Brownsville, Tex., as Mexican livestock inspector, supplanting Dr. Frank C. Elles, who has been transferred to the meat-inspection service at East St. Louis, Ill.

Dr. J. F. Jones, graduate of the Chicago Veterinary College, class of 1894, finds Arkansas City, Kan., a good location.

Dr. S. F. Luckey, graduate of Toronto, class of 1896, of Perryville, Mo., is a State inspector and located at Thayer, Mo. At this point a large dipping-vat will be completed in a short time, and all Southern cattle will be dipped for the purpose of destroying the ticks (*Boophilus bovis*), and thus in a measure controlling the disease. Dr. Rice P. Steddom will assist in the work at this point.

Dr. James C. Keeley, of Brownsville, Tex., graduate of the Kansas City Veterinary College, and recently located at the Chicago Station of the Bureau work, has been transferred to the quarantine division.

Drs. W. H. Richards and Louis F. Ryan are prominent practitioners at Emporia, Kan.

Dr. John T. Crosby, M.R.C.V.S., recently of St. Louis, Mo., finds his new location at Arkansas City, Kan., a promising one.

Dr. H. L. Ramacciotti, of Omaha, left on the 22d, with his wife and son, for a trip to Hot Springs and the Black Hills.

Dr. J. S. Anderson, of Seward, Neb., who was recently appointed State cattle inspector, has since enjoyed a great deal of riding over the State in search of the little tick, which he has succeeded in barring from the State. At his home he also enjoys a fine string of trotters, some of Greenlander's get.

Dr. J. E. Blackwell has left Omaha to take up his new position at St. Joe.

D. S. E. Cosford, of Omaha, has within the past year taken to himself a wife.

Dr. W. D. Hammond, of Wayne, Neb., the resident State Secretary of Nebraska for the U. S. V. M. A., has had his time well taken up gathering the reports on contagious diseases in that State.

Dr. J. J. Drasky, Crete, Neb., has shown the results of his extensive practice by building a fine new infirmary, thus showing the increasing tendency of making such investments in a State where it has been supposed that animals were too cheap to pay for treatment.

Dr. Fred Evans, who has recently located at Grand Island, Neb., reports a steadily increasing business.

Dr. V. Schaefer, of Tekamah, Neb., who is death on actinomycosis, has just completed a new infirmary and also made extensive additions to his house. He practically controls the practice of Burt County.

Dr. C. M. Day, of the Bureau of Animal Industry, is now stationed at the Nebraska Experiment Station, where he is coöperating in gathering data regarding serum as a cure and preventive for hog-cholera. He has made several trips over the State, making many friends for the Bureau.

Dr. George B. Tucker, of Lincoln, Neb., has completely renovated his large infirmary and made his office more spacious, indicating the rapid development of an extensive practice.

Dr. M. V. Byers, of Osceola, Neb., has the sympathy of his fellow-practitioners in the loss of his little child.

Dr. A. C. Bostrom, who has but recently located at Minden, Neb., has been making extensive investigations in "cornstalk disease" in horses and cattle.

---

### ANOTHER REMARKABLE LETTER.

deer Sir—I sit myself to ask you a profishonil Quistion—this is sumthing I sildum do as I am heed thority in this siction.

But We have a farmor who ouns a hurd of fiftine jursers an thore is Somthing wrong with thim *cattles* and I believe it is tubercoulocy--to Kiep up my rippution i must make a govnormental tist—whure can i git tubercolin—some body said Millcords are best and some one seys it is put under The hide with a haullow niddle—where can i git one and whare do you go under the hide at—i know the ballance of the manution bout the tist awnser quicke as you no it will take sum dais to git the wharewith.

Yourn Troughly

—— —— ——Dr of this siction.

Rats are often the carriers of contagious diseases from one locality to another. In China and India outbreaks of the bubonic plague have often been traced to this source.

All over the West the adoption of vaccination as the best solution for preventing outbreaks of anthrax and its allied forms has proved itself worthy of application, and is now relied upon by many who were at first doubtful of its efficacy.

